# Comparative Genomic Analysis and BTEX Degradation Pathways of a Thermotolerant *Cupriavidus cauae* PHS1

**DOI:** 10.4014/jmb.2301.01011

**Published:** 2023-04-10

**Authors:** Chandran Sathesh-Prabu, Jihoon Woo, Yuchan Kim, Suk Min Kim, Sun Bok Lee, Che Ok Jeon, Donghyuk Kim, Sung Kuk Lee

**Affiliations:** 1 School of Energy and Chemical Engineering, Ulsan National Institute of Science and Technology (UNIST), Ulsan 44919, Republic of Korea; 2 Department of Chemical Engineering, Pohang University of Science and Technology (POSTECH), Pohang, Gyeongbuk 37673, Republic of Korea; 3 Department of Life Science, Chung-Ang University, Seoul 06974, Republic of Korea

**Keywords:** BTEX, *Cupriavidus cauae*, degradation, genome analysis, thermotolerant

## Abstract

Volatile organic compounds such as benzene, toluene, ethylbenzene, and isomers of xylenes (BTEX) constitute a group of monoaromatic compounds that are found in petroleum and have been classified as priority pollutants. In this study, based on its newly sequenced genome, we reclassified the previously identified BTEX-degrading thermotolerant strain *Ralstonia* sp. PHS1 as *Cupriavidus cauae* PHS1. Also presented are the complete genome sequence of *C. cauae* PHS1, its annotation, species delineation, and a comparative analysis of the BTEX-degrading gene cluster. Moreover, we cloned and characterized the BTEX-degrading pathway genes in *C. cauae* PHS1, the BTEX-degrading gene cluster of which consists of two monooxygenases and meta-cleavage genes. A genome-wide investigation of the PHS1 coding sequence and the experimentally confirmed regioselectivity of the toluene monooxygenases and catechol 2,3-dioxygenase allowed us to reconstruct the BTEX degradation pathway. The degradation of BTEX begins with aromatic ring hydroxylation, followed by ring cleavage, and eventually enters the core carbon metabolism. The information provided here on the genome and BTEX-degrading pathway of the thermotolerant strain *C. cauae* PHS1 could be useful in constructing an efficient production host.

## Introduction

Soil and water contamination by petroleum hydrocarbons, including monoaromatic and polycyclic aromatic hydrocarbons, is a global environmental and health concern [1, 2]. Volatile organic compounds such as benzene, toluene, ethylbenzene, and three isomers of xylenes (BTEX) are the most common monoaromatic compounds in petroleum and have been classified as priority pollutants by the U.S. Environmental Protection Agency [3]. The high cost and inefficiency of physical and chemical methods for cleaning up contaminated sites have led to increased focus on BTEX removal by hydrocarbon-degrading bacteria through biodegradation and bioremediation, which are cost-effective, eco-friendly, and highly efficient biotechnology-based processes [4, 5]. Several microorganisms, including members of the genera *Pseudomonas*, *Ralstonia*, *Burkholderia*, *Sphingomonas*, *Thauera*, *Dechloromonas*, *Rhodococcus*, *Cladophialophora*, *Magnetospirillum*, *Pseudoxanthomonas*, *Comamonas*, *Bacillus*, *Microbacterium*, and *Acinetobacter*, have been reported to degrade all or some components of BTEX [6-14].

BTEX compounds are degraded through various metabolic pathways depending on the microorganism [12, 15, 16]. In general, aerobic degradation of BTEX begins with the incorporation of an oxygen atom and is catalyzed by monooxygenases or dioxygenases. The resulting catechol intermediates are then mineralized through either the ortho- or meta-cleavage pathway and catalyzed by catechol 1,2 dioxygenase or catechol 2,3 dioxygenase (C23O), respectively. This ultimately produces low-molecular mass compounds, such as aldehydes and pyruvate, which are further oxidized and incorporated into the metabolic pathways as sources of carbon and energy [10, 12, 16].

Monoaromatic hydrocarbon-degrading bacteria often have a broad range of substrate capabilities due to the multicomponent mono/di-oxygenases they express. For example, even though no gene encoding a benzene monooxygenase for the conversion of benzene to phenol was identified in *Paraburkholderia aromaticivorans* BN5, the toluene 4-monooxygenase (*tmoABCDE*) and phenol 2-hydroxylase (*dmpKLMNOP*) can catalyze the oxidation of benzene [15]. The naphthalene 1,2-dioxygenase of *Pseudomonas* sp. strain NCIB 9816-4 can convert all three xylene isomers into benzyl alcohols and aldehyde derivatives [17]. This low substrate specificity allows the bacteria to degrade related molecules through the same catabolic pathway. In contrast, the xylene monooxygenase of *P. putida* mt-2 or *B. amyloliquefaciens* subsp. *plantarum* strain W1 was found to only convert *p*-xylene and *m*-xylene, but not *o*-xylene [12, 18]. Generally, the three isomers of xylene are catabolized through the oxidation of a methyl group of xylene to 2-methylbenzyl alcohol by xylene monooxygenase or the direct oxidation of the aromatic ring by xylene dioxygenase [15, 19, 20].

Previously, we isolated and characterized the first BTEX-degrading thermotolerant bacterium, designated as PHS1, from a hot spring in Pohang, Korea. This bacterium, *Ralstonia* sp. PHS1, can grow efficiently on a variety of aromatic substrates at 35-45°C (optimally at 42°C) [8]. In mixtures of BTEX compounds, the strain could degrade all six BTEX components, with benzene being degraded the fastest, followed by toluene, *o*-xylene, ethylbenzene, and finally m- and *p*-xylene.

The *m*-xylene and *p*-xylene were found to be co-metabolized by strain PHS1 in the presence of the growth-supporting BTEX compounds [21]. The unique properties of strain PHS1, such as thermotolerance and the ability to degrade *o*-xylene, give the strain an advantage in the field of environmental restoration or for the treatment of hot industrial effluents contaminated with BTEX [8]. It is worth noting that among BTEX, *o*-xylene is the most recalcitrant compound in terms of microbial degradation, and only a limited number of microbial species capable of metabolizing *o*-xylene have been reported [5, 22, 23]. In addition, to fully grasp the physiological and genomic features of a particular strain, it is important that its complete genomic information be available. Therefore, in this study, we reclassified the PHS1 strain as *C. cauae* PHS1 and report its complete genome sequence, annotation, and species delineation along with a comparative analysis of the BTEX-degrading gene cluster, and proposed BTEX-degrading pathways.

## Materials and Methods

### Whole Genome Sequencing

To extract genomic DNA, strain PHS1 was cultured overnight in Luria-Bertani broth (LB; composition per liter: 5 g yeast extract, 10 g peptone, and 10 g NaCl) at 42°C with shaking at 200 rpm. The cells were collected by centrifugation at 12,000 ×*g* for 15 min at 4°C, and the LaboPass™ Genomic DNA Isolation Kit (Cosmogenetech Co., Ltd., Korea) was used to isolate the genomic DNA of PHS1.

For PacBio long-read genome sequencing library construction, 8 μg of gDNA was used, and the sequencing library was constructed using the PacBio DNA Template Prep Kit 1.0 (Pacific Biosciences, USA) following the manufacturer's instructions. The final SMRTbell library was sequenced using PacBio DNA Sequencing Kit 4.0 (Pacific Biosciences) and 8 SMRT cells with P6-C4 chemistry on the PacBio RS II sequencing platform (Pacific Biosciences). For the Illumina short-read library, 100 ng of gDNA was fragmented using a Covaris instrument (Covaris Inc., USA), and the library was prepared using a TruSeq Nano DNA High-Throughput Library Prep Kit (Illumina Inc., USA) according to the manufacturer's instructions. The library was then sequenced on the HiSeq platform (Illumina) after PCR amplification. The libraries were validated using an Agilent 2100 Bioanalyzer (Agilent Technologies, USA).

De novo assembly was performed using HGAP v3.0 based on PacBio reads, and gap-filling and error correction of the draft assemblies were performed using Pilon v1.21 with the aid of Illumina reads.

### Isolation of a BTEX-Degrading Gene Cluster and Plasmid Construction

All plasmids and strains used in this study are listed in Table S1. All DNA manipulations were performed in *Escherichia coli* TOP10 (USA). A genomic library was constructed by ligating partially Sau3AI-digested genomic DNA fragments of 5 to 15 kb from strain PHS1 into the pBluescript vector (pSK), which was digested with BamHI and treated with alkaline phosphatase. The DNA sequences were analyzed using Sanger DNA di-deoxy dye-terminator sequencing (Bionex Co., Ltd., Korea).

Transformants containing aromatic oxygenases were identified on LB agar plates containing 2 mM indole [24]. After incubating them for 3 days, the colonies were checked for the accumulation of blue pigment, which is a result of indigo formation. Two recombinant cells containing pSB1 or pSB2 were selected for further study. To analyze the initial ring-oxidation metabolites produced by BTEX monooxygenase 1 (Btxm1), pSB1-1 was generated by KpnI-digestion and self-ligation of pSB1 to eliminate beta-cleavage genes and prevent further reactions. pSB2 was used for functional analysis of BTEX monooxygenase 2 (Btxm2). All genes on these plasmids were expressed under the transcriptional control of the *lac* promoter in the pBluescript vector in *E. coli* TOP10.

### Metabolite Isolation and Identification

*E. coli* cells containing pSB1-1 or pSB2 grown in LB medium were induced with 100 μM IPTG for 6 h, and then washed twice in 0.1 M potassium phosphate buffer (pH 7.0). The cells were resuspended in the same buffer and supplemented with 1.5 mM toluene, and then shaken at 37°C for 2 h. The metabolite extraction and gas chromatography analysis were performed using a previously described procedure [8]. Metabolites produced from the oxidation of toluene were identified by comparing their retention times with those of authentic samples.

### Genomic Analysis

The genomic content of strain PHS1 was annotated based on the COG database (cog-20), UniProt (Swiss-Prot, TrEMBL), BRENDA, and KEGG using DIAMOND v2.0.11 [25-28] and BlastKOALA [29]. The subjects with the highest *e*-value, as well as identity and query coverage over 50%, were used for analysis based on the BLAST (DIAMOND) results. To predict genomic islands in the PHS1 genome, IslandViewer 4 was used with three methods: IslandPath-DIMOB, SIGI-HMM, and IslandPick [30].

To find gene candidates that may be involved in BTEX degradation, sequences of proteins involved in aromatics degradation were collected from the KEGG orthology and NCBI database. The collected sequences included: xylene monooxygenase, benzoate (toluate) 1,2-dioxygenase, benzylalcohol dehydrogenase, benzaldehyde dehydrogenase, 4-hydroxybenzaldehyde dehydrogenase, 4-cresol dehydrogenase, 4-hydroxybenzoate dehydrogenase, phenylpropionate dehydrogenase, dihydroxycyclohexadiene carboxylate dehydrogenase, naphthalene dioxygenase, ethylbenzene dioxygenase, ethylbenzene hydroxylase, toluene methyl-monooxygenase, aryl-alcohol dehydrogenase, phenol 2-monooxygenase, and benzene/toluene/chlorobenzene dioxygenase. The collected protein sequences were compared with all the coding sequences of the strain using NCBI BLAST+ 2.11.0.

### Phylogenetic Analysis

For phylogenetic analysis, the 16S rRNA gene sequences of strain PHS1 and *Cupriavidus* representative strains were extracted from their corresponding whole genome sequences based on gene annotations. The 16S rRNA gene sequences of the PHS1 strain and closely related type strains were aligned using the fast secondary-structure aware infernal aligner available at the Ribosomal Database Project (https://pyro.cme.msu.edu/aligner/form.spr). A phylogenetic tree based on the 16S rRNA gene sequences was constructed using the neighbour-joining algorithm with bootstrap values (1,000 replications) using MEGA11 software [31]. The 16S rRNA gene sequence similarity values between the PHS1 strain and closely related type strains were calculated using the Nucleotide Similarity Search program on the EzBioCloud server (www.ezbiocloud.net/identify). For the genome-based phylogenetic analysis, the up-to-date bacterial core gene pipeline (UBCG; https://help.ezbiocloud.net/ubcg-gene-set/) (Kim *et al*., 2021) was used to extract 92 core genes from the genomes of the PHS1 strain and closely related type strains, which were then concatenated and aligned. A maximum-likelihood tree based on the concatenated nucleotide sequences of the 92 core genes was reconstructed using MEGA11 software.

The average nucleotide identity (ANI) and digital DNA-DNA hybridization (DDH) values between the PHS1 strain and closely related type strains were calculated using the Orthologous Average Nucleotide Identity Tool (OAT) software available on the EzBioCloud webserver (www.ezbiocloud.net/sw/oat) [33] and the server-based Genome-to-Genome Distance Calculator version 2.1 (http://ggdc.dsmz.de/distcalc2.php) [34], respectively.

To construct a single-gene tree of bacterial monooxygenase alpha subunits, pairwise alignments of collected protein sequences from reference articles were carried out using MUSCLE built in MEGA 11 with the default option. The gene tree was constructed using the maximum-likelihood method with the Jones-Taylor-Thornton (JTT) model and 1,000 iterations [31].

## Results and Discussion

### Reclassification and Species Delineation of the PHS1 Strain

Previously, strain PHS1 was identified as belonging to the genus *Ralstonia* [8]. However, the 16S rRNA gene sequence of PHS1 was re-examined in the present study using various computational tools and algorithms. The 16S rRNA gene sequence of strain PHS1 and closely related type strains were aligned using the fast secondary-structure aware infernal aligner available at the Ribosomal Database Project (https://pyro.cme.msu.edu/aligner/form.spr). A phylogenetic tree based on 16S rRNA gene sequences, constructed using the neighbour-joining algorithm, showed that strain PHS1 was not a species of *Ralstonia* (Fig. S1).

**Fig. 1:**
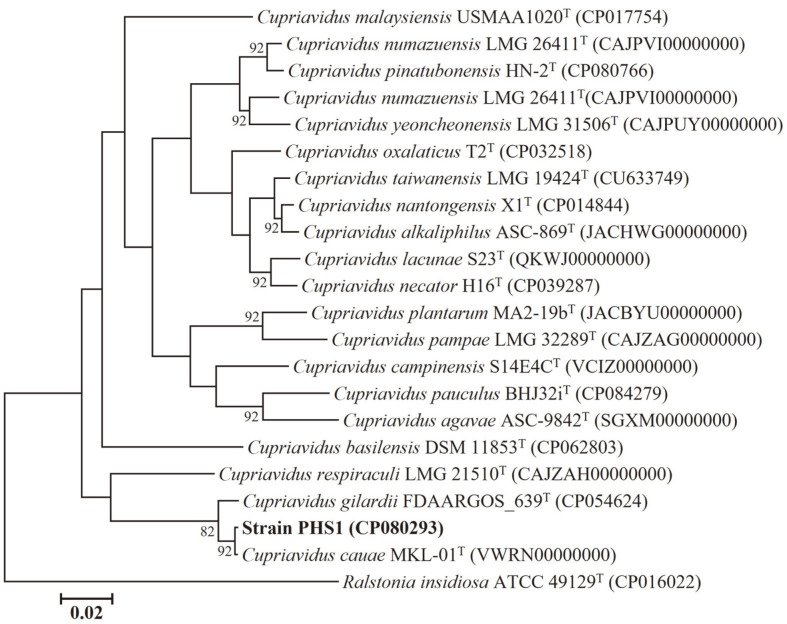
Phylogenomic tree based on the concatenated 92 core genes showing the phylogenetic relationships between strain PHS1 and closely related taxa. Numbers on nodes correspond to bootstrap values for branches (1,000 replicates), shown only bootstrap values over 70%. *R. insidiosa* AU2944 was used as the outgroup. Bar, 0.02 substitutions per nucleotide.

For genome-based phylogenetic analysis, a maximum-likelihood tree showing the phylogenetic relationships between strain PHS1 and closely related taxa was constructed (Fig. 1). The highest sequence similarity was observed with species belonging to the genus *Cupriavidus*.

Furthermore, the average nucleotide identity (ANI) and digital DNA-DNA hybridization (DDH) values between strain PHS1 and closely related type strains were calculated [33,34]. Strain PHS1 showed very high similarities with *C. cauae* MKL-01 and *C. gilardii* FDAARGOS_639, with 99.87% and 99.74% 16S rRNA similarities and 98.33% and 91.33% ANI values, respectively (Table 1). Although the ANI value of *C. cauae* was much higher than that of *C. gilardii*, there was no significant difference observed in 16S rRNA similarities. Considering the possibility that strain PHS1 may be closer to other strains of *C. gilardii*, further comparisons were carried out using ANI and DDH with all strains of *C. cauae* (1) and *C. gilardii* (10) deposited in the NCBI assembly database (Table S2). Most comparisons with *C. gilardii* strains showed low ANI and DDH values below the threshold for delineating the same species (ANI: 95%; DDH: 70%) [35], except for the second highest values for *C. gilardii* W2-2 with 98.16% and 85.5% values, respectively (Table S2).

The ANI and digital DDH values between strain PHS1 and *C. cauae* MKL-01, the most closely related type strain, were less than 98.3% and 86.0%, respectively. This suggests that strain PHS1 represents a strain member of *C. cauae*.

### General Characteristics of the PHS1 Genome

**Fig. 2:**
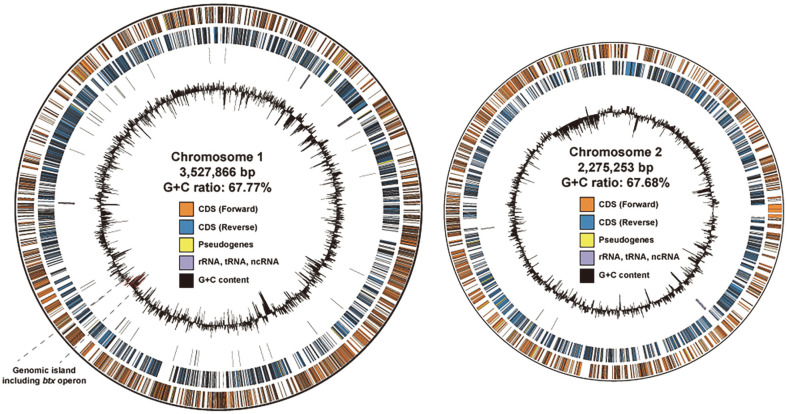
Genomic characteristics of strain PHS1. Circular visualization of two chromosomes in strain PHS1. Orange and blue tiles indicate the CDS with forward and reverse direction. Yellow tiles indicate pseudogenes. Purple tiles indicate noncoding RNA including rRNA and tRNA. Black histogram indicates the GC contents of each coding sequence. The genomic island including the *btx* operon is represented as dotted lines and red histogram. Data were visualized using Circos [76].

The genomic features of strain PHS1 are summarized in Table 2. The genome contains two circular chromosomes, with a total of 5,075 genes (Fig. 2). This is similar to the genome structure of *Cupriavidus*, which also has two circular chromosomes [36]. The sizes of the chromosomes in strain PHS1 are 3,527,866 bp (3,120 genes) and 2,275,253 bp (1,955 genes), respectively. Additionally, 126 pseudogenes, 4 complete rRNA clusters, and 57 tRNA genes were predicted from annotation by the NCBI PGAP pipeline. Of these, 3 complete rRNA clusters and 51 tRNA genes are located on chromosome 1. The average GC content of the PHS1 genome is 67.73%, with similar GC content for each chromosome (67.77% and 67.68%, respectively). Genomic islands were also predicted using IslandViewer4 and a total of 52 were found in the PHS1 genome.

Clusters of orthologous genes (COG) analysis was conducted on each genome to investigate the distribution of genes in each chromosome. The predicted CDS of the PHS1 genome were compared with protein sequences in the COG database [27]. Among the 26 categories in the COG database, genes involved in 11 categories (J, L, D, V, M, N, W, U, O, F, and H) were unevenly distributed across the two chromosomes (Fig. S2). In other categories, such as K (transcription) or C (energy production and conversion), the related genes were evenly distributed across the two chromosomes. These distributions and the conservation of the two circular chromosomes suggest that they may play complementary roles in various aspects of cellular functions, not just metabolism. The complete genome information for strain PHS1 has been deposited in NCBI under the GenBank assembly accession number GCA_026210475.1 (https://www.ncbi.nlm.nih.gov/assembly/GCA_026210475.1).

### Comparative Analysis of the BTEX-Degrading Gene Cluster

**Fig. 3:**
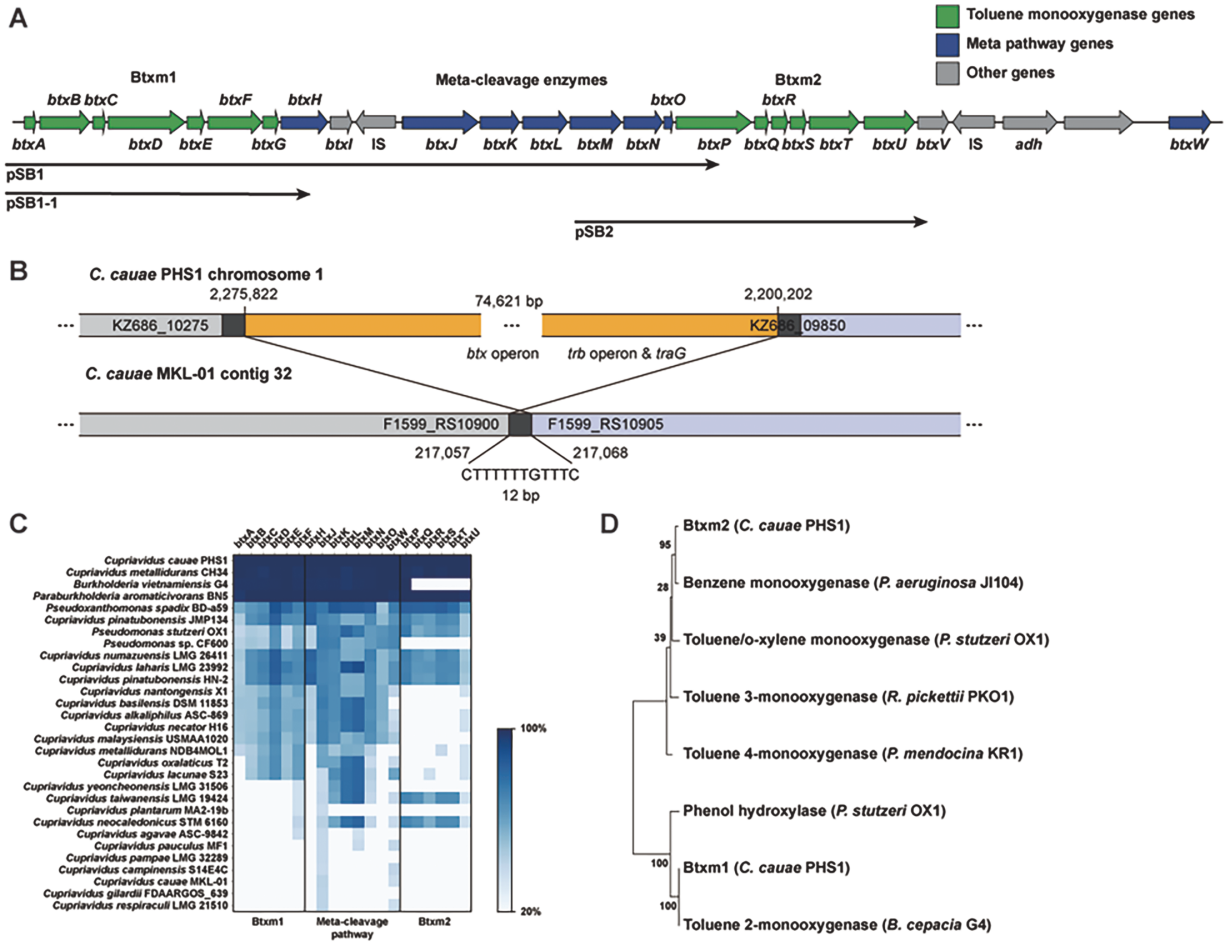
Gene arrangements and comparison of genes in BTEX-degrading gene cluster. (**A**) Genes encoding two toluene monooxygenase (green) and meta-cleavage pathway (blue) were adjacently located in chromosome 1 of strain PHS1 genome. Screened plasmids, pSB1, pSB1-1, and pSB2, are indicated as black arrow. IS denotes insertion sequence. (**B**) The integration site of the approximately 75 kbp genetic fragment containing the *btx* operon. The nucleotide sequences flanking the insertion sequence in PHS1 were almost identical to those in MKL-01, except for 12 bp duplicated sequences. (**C**) Protein sequences of genes in BTEX-degrading gene cluster are compared with *Cupravidus* representative strains and several strains reported as degraders of aromatics. (**D**) Protein sequence tree of known aromatic monooxygenases subunit α were reconstructed based on multiple sequence alignments. Numbers at nodes indicate the percentage of node resampled.

The BTEX-degrading gene cluster (BDGC) identified in the genome of strain PHS1 was located at the position 2,217,391 - 2,240,837 in chromosome 1. We found it consisted of genes encoding two monooxygenases, Btxm1 and Btxm2, as well as enzymes involved in the meta-cleavage pathway (Fig. 3A). The functions of these genes were predicted bioinformatically and are summarized in Table 3. The genomic DNA library of strain PHS1 was screened to clone the genes for BTEX catabolism using the enzymatic oxidation of indole to indigo by aromatic oxygenases [37]. Two types of aromatic oxygenases with different characteristics were isolated from the genomic library. In particular, the accumulation of a ring fission product [38], indicated by a yellow color, was observed in *E. coli* TOP10 cell cultures harboring the pSB1 plasmid (Fig. 3A) when exposed to toluene. This indicates that the pSB1 insert codes for enzymes are involved in the transformation of toluene into the corresponding meta-cleavage products catalyzed by C23O [39]. The other type of oxygenase was found in the strain TOP10 harboring pSB2 (Fig. 3A). These findings suggest that strain PHS1 has the ability to efficiently degrade BTEX compounds through the meta-cleavage pathway, using the Btxm1 and Btxm2 monooxygenases and the associated enzymes.

After comparing the nucleotide sequences of the BDGC with representative strains of *Cupriavidus*, we found that *C. metallidurans* CH34 showed full conservation of the BDGC (Fig. 3C). Additionally, analysis of the BDGC with other genera known to be involved in aromatic degradation revealed that the Btxm1 and meta-cleavage pathway of *B. vietnamiensis* G4 and *Pseudomonas* sp. CF600 are closely related and homologous to the BDGC. The BDGC (comprising Btxm1, Btxm2, and the meta-cleavage pathway) was also conserved in *P. aromaticivorans* BN5, with nearly 100% similarity at the nucleotide level (Fig. 3C) [15, 40-45]. Interestingly, no genes in the BDGC were conserved in closely related strains, such as *C. cauae* and *C. gilardii*, but were rather conserved in phylogenetically distant strains. Additionally, the GC content of the BDGC (57.5%) was significantly lower than that of the genome (67.73%). It was also predicted that the BDGC is a genomic island using IslandPath-DIMOB, SIGI-HMM, and IslandPick algorithms in the IslandsViewer 4 software (Fig. 2) [30]. These findings suggest that the BDGC in strain PHS1 was likely acquired horizontally rather than being conserved across genera or species.

To determine whether any genes other than the BDGC may be involved in the biodegradation of BTEX compounds, the entire PHS1 genome was annotated using various databases, including UniProt, KEGG, and BRENDA (Table S3). Then, all hydroxylases and oxygenases that might be involved in the early stages of BTEX degradation were investigated. As a result, none of the genes were found to be potentially involved in BTEX degradation, except for KZ18760-KZ18775, which were predicted to be dioxygenases for aromatic compounds [46-48].

In addition to analyzing the BDGC, the protein orthologous sequences known to be involved in the early stages (before cleavage of the aromatic ring) of BTEX degradation were collected and compared to the protein sequences of the PHS1 strain genome using BLAST [49]. For example, a total of 281 orthologous sequences of *p*-cresol dehydrogenase (*pchCF*), which catalyzes the methyl group of *p*-cresol (a derivative of toluene) to yield p-hydroxybenzylaldehyde, were collected. The BLAST results showed that the highest match of *pchF* orthologs had only 27.1% sequence similarity with the PHS1 genomic content, and none of the protein sequences in strain PHS1 genome were aligned with *pchC* orthologs. This suggests that the PHS1 strain does not encode *p*-cresol dehydrogenase in its genome. Following an examination of the gene candidates that were listed in the Materials and Methods section as potential contributors to BTEX degradation, we found that strain PHS1 lacks any known enzymes that support the early stages of BTEX degradation, except for those found in the *btx* operon (as shown in Table S4). As such, we have concluded that the PHS1 strain does not harbor any additional gene candidates involved in BTEX degradation.

To further investigate the differences between the *C. cauae* PHS1 and *C. cauae* MKL-01 genomes, the coding sequences from both strains were compared based on an ortholog search. A total of 4,369 orthologous genes were identified between the two genomes using the Reciprocal Best Hit method with a Smith-Waterman algorithm, with a 50% identity and 80% coverage threshold in both directions [50]. A total of 508 and 511 non-orthologous genes were respectively identified in PHS1 and MKL-01 (Fig. S3). The BTEX-degrading operon (*btx* operon) was not present in MKL-01. Most of the genes within the *btx* operon were classified as COG category Q (secondary metabolites biosynthesis, transport, and catabolism). The number of COG category X genes present in PHS1, which are indicative of the mobilome involved in horizontal gene transfer, was 25, a number greater than the 13 found in MKL-01. Additionally, we identified 12 COG category U genes in PHS1 that are associated with intracellular trafficking, secretion, and vesicular transport. Of these 12 genes, 11 were located adjacent to the *btx* operon. Notably, 10 of these genes upstream of the *btx* operon appeared to form the *trb* operon and *traG* gene, which are known to play a crucial role in facilitating the conjugative transfer of plasmids (KZ686_09850, KZ686_09855, KZ686_09860, KZ686_09865, KZ686_09875, KZ686_09880, KZ686_09885, KZ686_09890, KZ686_09895, and KZ686_09905) [51]. These findings suggest that horizontal gene transfer may occur in PHS1, as shown in Figs. S2 and 3B. The integration site of the *btx* operon in MKL-01 was analyzed by comparing the conserved nucleotide sequences of the flanking region with those of the strain PHS1. The genomic island containing the *btx* operon was 74,621 bp and may have been integrated into the 217,057 to 217,068 region of contig 32 of the MKL-01 genome (an incompletely assembled genome). The flanking genes of the MKL-01 strain were F1599_RS10900 and F1599_RS10905 (Fig. S3B).

### BTEX-Monooxygenase 1 (Btxm1)

Analysis of the nucleotide sequences of the Btxm1 genes (*btxABCDEF*) showed that they were closely related in gene order and homologous to a group of T2MOs from *Burkholderia* sp. JS150 and *B. cepacia* G4, and phenol hydroxylases (PH) from *C. testosteroni* TA441, *P. stutzeri* OX1, and *P. putida* CF600 [52-55] (Fig. 3D). Specifically, BtxABCDEF showed high overall sequence identity (> 98%) to TomABCDEF from the approximately 108-kb degradative plasmid TOM of *B. cepacia* G4, which is known to metabolize *o*-xylene as a carbon source [56].

BtxD, the largest polypeptide of Btxm1, was homologous to the large oxygenase subunit of other bacterial multicomponent monooxygenases (BMM) containing two dinuclear iron-binding domains with the amino acid sequence Asp-Glu-X-Arg-His, which is found in several enzymes that catalyze reactions involving activated oxygen [57]. BtxB shares homology with the small oxygenase subunits of PHs and TMOs. BtxF is homologous to a number of other bacterial iron-sulfur flavoproteins that serve as oxidoreductases for several enzyme systems, including monooxygenases, aromatic dioxygenases, and reductases involved in the biosynthesis of deoxy-sugars [58]. Database comparisons using the deduced amino acid sequences of *btxA*, *btxC*, and *btxE* as query sequences showed that each of these three gene products has homologous counterparts in PHs and T2MOs.

### Meta-Cleavage Pathway

The meta-pathway genes (*btxGHIJKLMNO*) are closely related to the corresponding phenol-degrading *aph* and *dmp* genes of the strains *C. testosteroni* TA441 and *Pseudomonas* sp. CF600, respectively (Fig. 3). In particular, the meta-pathway gene products of *Burkholderia* sp. RP007 that degrade polycyclic aromatic hydrocarbons (PAHs), PhnT2/E2/X, showed 95% overall sequence identity to BtxG/H/I, respectively. BtxG, which is homologous to ferredoxins, contains the amino acid motif Cys-XXXX-Cys-XX-Cys that is characteristic of chloroplast-type ferredoxins [59]. In addition to PhnE2 of *Burkholderia* sp. RP007, the PHS1 C23O (*btxH*) has 97% identity with TomB from *o*-xylene-degrading *B. cepacia* G4. A fragment encoding an ORF of 149 amino acids was found downstream of C23O, which is preceded by a Shine-Dalgarno-like sequence, AGGAGA. The predicted amino acid sequence of this ORF shares sequence identity with an unknown protein and has no assigned function that is associated with other catabolic gene clusters, including meta-cleavage genes in *Burkholderia* sp. RP007 [60], *C. testosteroni* TA441 [61], and *Sphingomonas* sp. HV3 [13]. A putative 2-hydroxymuconic semialdehyde dehydrogenase (BtxJ) gene is located downstream of the insertion sequence (IS), starting 23 bp downstream of the end of the IS. The *btxJ* is followed by the genes *btxKLM*, which encode 2-hydroxypent-2,4-dienoate hydratase, acetaldehyde dehydrogenase, and 4-hydroxy-2-oxovalerate aldolase, respectively. These enzymes catalyze the final three steps of the meta-pathway that are common to other meta-degradation pathways for various aromatic compounds. The genes for the 4-oxalocrotonate branch enzymes, 4-oxalocrotonate decarboxylase (BtxN) and 4-oxalocrotonate tautomerase (BtxO), follow BtxM [62].

Most meta-cleavage pathways can be classified into two types [60]: P-type (gene order: Fn, C23O, HMSD, HMSH) from *Pseudomonas* spp. [44] and S-type (gene order: GST, HMSH, C23O, U, HMSD) from *Sphingomonas* spp. [13,63]. However, the meta-pathway of strain PHS1 (gene order: Fn, C23O, U, HMSD) is a mixture of both types, similar to the PAH-degrading pathway of *Burkholderia* RP007 [60]. Homologous polypeptides were also found in other enzymes, particularly in the phenol-degrading *aph* and *dmp* systems of *C. testosteroni* TA441 and *Pseudomonas* sp. strain CF600, respectively, and the PCB-degrading *bph* system of *Pseudomonas* sp. strain LB400 [64]. The hydrolytic branch involving the enzyme HMSH (BtxW), which corresponds to DmpD in strain CF600, is required for the degradation of 2- and/or 3-methylated phenols [44]. However, the gene for HMSH was not found in the *aph* and *btx* gene clusters of strain TA441 and PHS1, respectively [61]. Unlike strain TA441, strain PHS1 is able to utilize various methylphenols such as *o*-, *m*-, *p*-cresol, and 2,3-dimethylphenol [8]. This suggests that the gene for HMSH may exist downstream of the GST gene, as in the S-type meta-pathway (GST-HMSH-C23O-U-HMSD) [60]. In strain PHS1, the HMSH gene is immediately downstream of the *btx* cluster and designated as KZ686_10070, with over 70% similarity to the HMSH gene of strain CF600. In *C metallidurans* CH34 (GenBank accession no. NC 007973), the HMSH gene is located downstream of the GST gene.

### BTEX-Monooxygenase 2 (Btxm2)

The Btxm2 enzyme is composed of BtxPQRSTU and is similar to toluene/*o*-xylene monooxygenase (ToMO). BtxP and BtxT are similar to the large and small oxygenase subunits, respectively. Like BtxD in Btxm1, BtxP contains a pair of dinuclear iron-binding ligands with the amino acid sequence Asp-Glu-X-Arg-His between amino acids 133 and 137, and 230 and 234. BtxU is similar to the NADH-ferredoxin oxidoreductase components of several mono- and dioxygenase systems. BtxS is a coupling/effector protein that affects the activity and specificity of other bacterial oxygenase systems [65]. BtxR has a region between amino acids 40 and 70 that can be aligned with a domain found in many proteins classified as ferredoxin types. In addition, two conserved Cys residues followed by His residues (Cys45, His47, and Cys64, His67 in BtxR), which are believed to be involved in coordinating a Rieske-type iron-sulfur cluster, are present [66].

Based on multiple sequence alignments of the 8 alpha subunits of known TMOs, an evolutionary tree of protein sequences was constructed (Fig. 3D). Btxm2 is similar to the benzene monooxygenase from *P. aeruginosa* JI104, ToMO from *P. stutzeri* OX1, toluene 3-monooxygenase (T3MO) from *R. pickettii* PKO1, and toluene 4-monooxygenase (T4MO) from *P. mendocina* KR1 [67-70].

### Proposed BTEX Biodegradation Pathways in PHS1

Previously, we reported that the main products of benzene and *o*-xylene degradation by strain PHS1 were phenol and 2,3-dimethylphenol, respectively, based on mass spectrometric analysis [8]. These observations suggest that the enzyme involved in the first step of individual BTEX degradation directly mono-oxidizes the aromatic nucleus [71, 72].

**Fig. 4:**
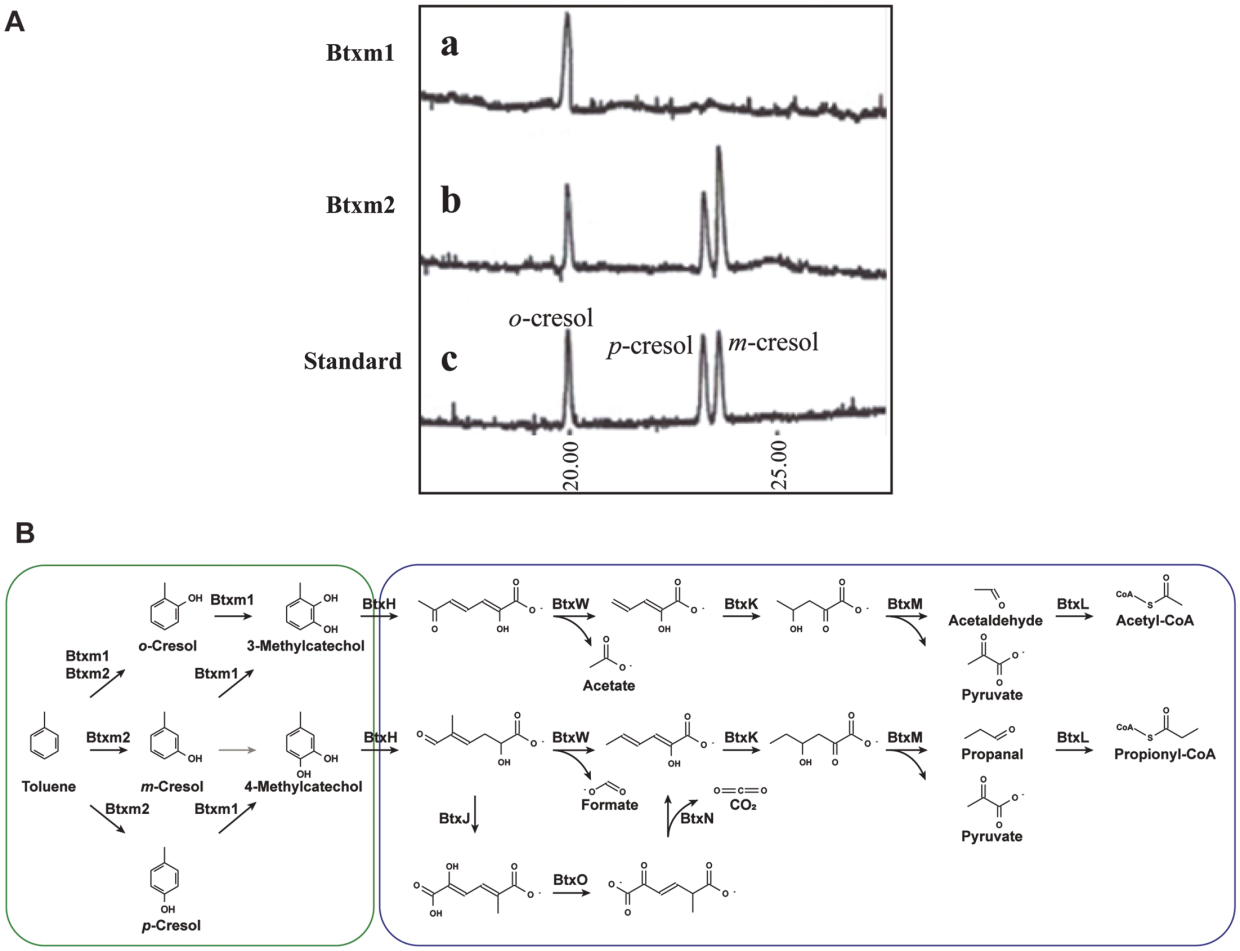
Proposed BTEX- biodegradation pathways in strain PHS1. (**A**) Gas chromatographs of metabolites produced from toluene by cells of TOP10. (a) Btxm1, (b) Btxm2, (c) standards (in order of *o*-, *p*-, *m*-cresol). (**B**) Reactions in green outline indicate the catalytic reactions of two toluene monooxygenase, while those in blue outline indicate the reactions catalyzed by meta-cleavage enzymes.

Toluene has been studied as a representative model compound of monoaromatic hydrocarbons [73]. Btxm1 only produced o-cresol from toluene (Fig. 4A), indicating that its activity is similar to that of the multicomponent T2MO from *B. cepacia* G4 [52] and *B. cepacia* JS150 [74]. In contrast, Btxm2 produced *o*-, *m*-, and *p*-cresol compounds (Fig. 4A), indicating that Btxm2 has low regioselectivity, similar to the benzene monooxygenase of *P. aeruginosa* JI104 [67] and ToMo of *Pseudomonas* sp. OX1 [24], which can produce three cresol compounds from toluene. The three isomers of cresols may then be further converted into 3-methylcatechol or 4-methylcatechol by Btxm1 or Btxm2 activity. The phenol hydroxylase (PH) from *P. stutzeri* OX1 is known to have catalytic activity for converting these three isomers of cresols into 3-methlycatechol (from *o*-cresol and *m*-cresol) and 4-methylcatechol (from *p*-cresol) [38]. Next, 3- or 4-methylcatechol may be cleaved by catechol 2,3-dioxygenase (C23O) to yield ring cleavage products, 2-Hydroxy-6-oxo-hept-2,4-dienoate or (2E,4Z)-2-Hydroxy-5-methyl-6-oxohexa-2,4-dienoate, depending on the substrate specificity of C23O of *P. stutzeri* OX1 [75]. Finally, the intermediates may be degraded by BtxIJKLMNO to yield pyruvate and acetyl-CoA, which are universally utilizable metabolites that enter the central carbon metabolism (Fig. 4B).

In the case of ethylbenzene degradation, it is likely to be initiated by Btxm2, considering the ethylbenzene selectivity of BMO from *P. aeruginosa* JI104, ToMO from *P. stutzeri* OX1, and T3MO from *R. pickettii* PKO1 [24, 67, 68]. The main products reported from ethylbenzene degradation are 3-ethylphenol (produced by BMO, ToMO, and T3MO) and 4-ethylphenol (produced by T3MO). Therefore, considering the phylogenetic relationship of toluene monooxygenases, it is likely that the degradation of ethylbenzene in strain PHS1 begins with the aromatic ring hydroxylation rather than the hydroxylation of the ethyl group by Btxm2.

The two toluene monooxygenases (Btxm1 and Btxm2) in the BDGC of strain PHS1 may catalyze the hydroxylation of aromatics, but they are expected to have different specificities for non-hydroxylated and monohydroxylated compounds, as well as regioselectivity related to the location of the methyl group. In *P. stutzeri* OX1, the K_cat_/K_m_ value for benzene with ToMO is much larger than that of phenol, while PH is much more effective at converting phenol than benzene [53]. In addition, PH can act on various monohydroxylated aromatics, such as cresol and dimethylphenol [38]. It appears that Btxm1 and Btxm2 also catalyze different steps in the two hydroxylations of aromatics in a complementary manner. PH mainly catalyzes the second hydroxylation of toluene and xylene derivatives, as well as benzene, rather than the first one [38,53]. Therefore, it is speculated that Btxm2 mainly catalyzes the first hydroxylation of various aromatic compounds with low regioselectivity, and Btxm1 mainly catalyzes the second hydroxylation at the ortho-position of phenolic compounds, as well as the first hydroxylation at the ortho-position of toluene.

In conclusion, our results suggest that new catabolic pathways may have arisen through enzyme structural evolution to allow for a wider range of substrates or the acquisition of catabolic functions from different bacteria, in order to promote rapid adaptation to diverse environmental conditions. The BDGC of PHS1, comprising the two TMOs and the meta-pathway, is thought to have an efficient architecture with complementary activities of the two TMOs and cooperating meta-cleavage enzymes, as well as the advantage of a clustered structure of gene arrangements. Further analysis revealed that the BDGC is conserved in certain related strains but not in others, suggesting that it may have been acquired horizontally rather than being conserved across genera or species.

## Supplemental Materials

Supplementary data for this paper are available on-line only at http://jmb.or.kr.

## Figures and Tables

**Table 1:** 16S rRNA and ANI comparison of *Cupriavidus* representative strains with strain PHS1.

Strains	16S rRNA BLAST identity	16S rRNA BLAST coverage	ANI value*
*C. cauae* MKL-01	99.87	99	98.33
*C. gilardii* FDAARGOS_639	99.74	99	91.33
*C. nantongensis* X1	98.76	99	82.72
*C. oxalaticus* T2	98.44	99	82.50
*C. respiraculi* LMG 21510	98.76	99	82.31
*C. neocaledonicus* STM 6160	98.56	99	81.91
*C. taiwanensis* LMG 19424	98.83	99	81.80
*C. alkaliphilus* ASC-869	NA	NA	81.79
*C. necator* H16	98.37	99	81.70
*C. lacunae* S23	NA	NA	81.68
*C. campinensis* S14E4C	98.30	100	81.58
*C. malaysiensis* USMAA1020	98.70	100	81.34
*C. laharis* LMG 23992	98.05	99	81.23
*C. yeoncheonensis* LMG 31506	NA	NA	81.03
*C. basilensis* DSM 11853	97.98	99	81.00
*C. numazuensis* LMG 26411	NA	NA	80.82
*C. agavae* ASC-9842	98.24	99	80.79
*C. pinatubonensis* HN-2	98.24	100	80.46
*C. pauculus* MF1	98.89	99	80.40
*C. metallidurans* NDB4MOL1	98.89	100	80.24
*C. pampae* LMG 32289	98.50	100	79.92
*C. plantarum* MA2-19b	99.22	100	79.82

*Average nucleotide identity; All the values are expressed as a percentage (%).

NA, not available.

**Table 2:** General characteristics of the PHS1 strain genome.

Feature	Chromosome 1	Chromosome 2	Total
Bioproject no.	PRJNA749683		
Biosample no.	SAMN20397041		
GenBank accession no.	GCA_026210475.1		
Size (bp)	3,527,866	2,275,253	5,803,119
G+C content (%)	67.77	67.68	67.73
Total genes	3,120	1,955	5,075
Protein coding sequences	3,057	1,946	4,877
Pseudogenes	76	50	126
Complete rRNAs	3	1	4
tRNA genes	51	6	57
ncRNA, regulatory, etc.	3	0	3
Genomic islands	31	21	52

**Table 3:** Gene contents of BTEX-degrading gene cluster.

Locus tag	Gene	Location	Strand	% G+C	Length	Product
KZ686_09940	*btxA*	2217391-2217615	+	55.56	74	Phenol hydroxylase subunit (PH)
KZ686_09945	*btxB*	2217703-2218698	+	56.63	331	Phenol hydroxylase subunit (β)
KZ686_09950	*btxC*	2218738-2219007	+	51.85	89	Phenol hydroxylase subunit
KZ686_09955	*btxD*	2219030-2220589	+	55.19	519	Phenol hydroxylase subunit (α)
KZ686_09960	*btxE*	2220586-2220942	+	58.82	118	Phenol hydroxylase subunit (γ)
KZ686_09965	*btxF*	2221021-2222085	+	57.37	354	Phenol hydroxylase subunit
KZ686_09970	*btxG*	2222088-2222444	+	59.38	118	Ferredoxin (Fn)
KZ686_09975	*btxH*	2222462-2223406	+	55.87	314	Catechol 2,3-dioxygenase (C23O)
KZ686_09980	*btxI*	2223428-2223877	+	61.78	149	Unknown protein
KZ686_09985	*IS*	2223931-2224748	-	61.37		IS element family transposase
KZ686_09990	*btxJ*	2224862-2226373	+	63.29	503	2-Hydroxymuconic semialdehyde dehydrogenase (HMSD)
KZ686_09995	*btxK*	2226376-2227158	+	61.56	260	2-hydroxypent-2,4-dienoate hydratase (OEH)
KZ686_10000	*btxL*	2227237-2228148	+	61.07	303	Acetaldehyde dehydrogenase (ADA)
KZ686_10005	*btxM*	2228168-2229214	+	62.18	348	4-Hydroxy-2-oxovalerate aldolase (HOA)
KZ686_10010	*btxN*	2229211-2229999	+	62.86	262	4-oxalocrotonate decarboxylase (4OD)
KZ686_10015	*btxO*	2230011-2230202	+	59.38	63	4-Oxalocrotonate tautomerase (4OT)
KZ686_10020	*btxP*	2230262-2231764	+	57.49	500	Toluene monooxygenase subunit (α) (TMO)
KZ686_10025	*btxQ*	2231833-2232099	+	58.43	88	Toluene monooxygenase subunit (γ)
KZ686_10030	*btxR*	2232155-2232490	+	54.46	111	Ferredoxin
KZ686_10035	*btxS*	2232531-2232845	+	56.83	104	Toluene monooxygenase subunit
KZ686_10040	*btxT*	2232900-2233886	+	56.33	328	Toluene monooxygenase subunit (β)
KZ686_10045	*btxU*	2233986-2235008	+	55.33	340	Toluene monooxygenase subunit
KZ686_10050	*btxV*	2235074-2235679	+	53.14	201	Glutathione S-transferase (GST)
KZ686_10055	*IS*	2235750-2236567	-	60.27		IS element family transposase
KZ686_10060	*adh*	2236737-2237798	+	55.08	353	Alcohol dehydrogenase
KZ686_10065		2237943-2239271	+	61.63	442	Putative transporter
KZ686_10070	*btxW*	2240007-2240837	+	56.92	276	2-Hydroxymuconate-semialdehyde hydrolase (HMSH)
