# Supplementary material for: Comparative Genomic Analysis and BTEX Degradation Pathways of a Thermotolerant Cupriavidus cauae PHS1
Source: J Microbiol Biotechnol. 2023 Apr 10;33(7):875–85. doi: 10.4014/jmb.2301.01011 (PMC10394343; doi:10.4014/jmb.2301.01011)
Supplement: Supplementary file 1 [file jmb-33-7-875-supple.pdf]

https://doi.org/10.4014/jmb.2301.01011

Supplementary Figures and Table

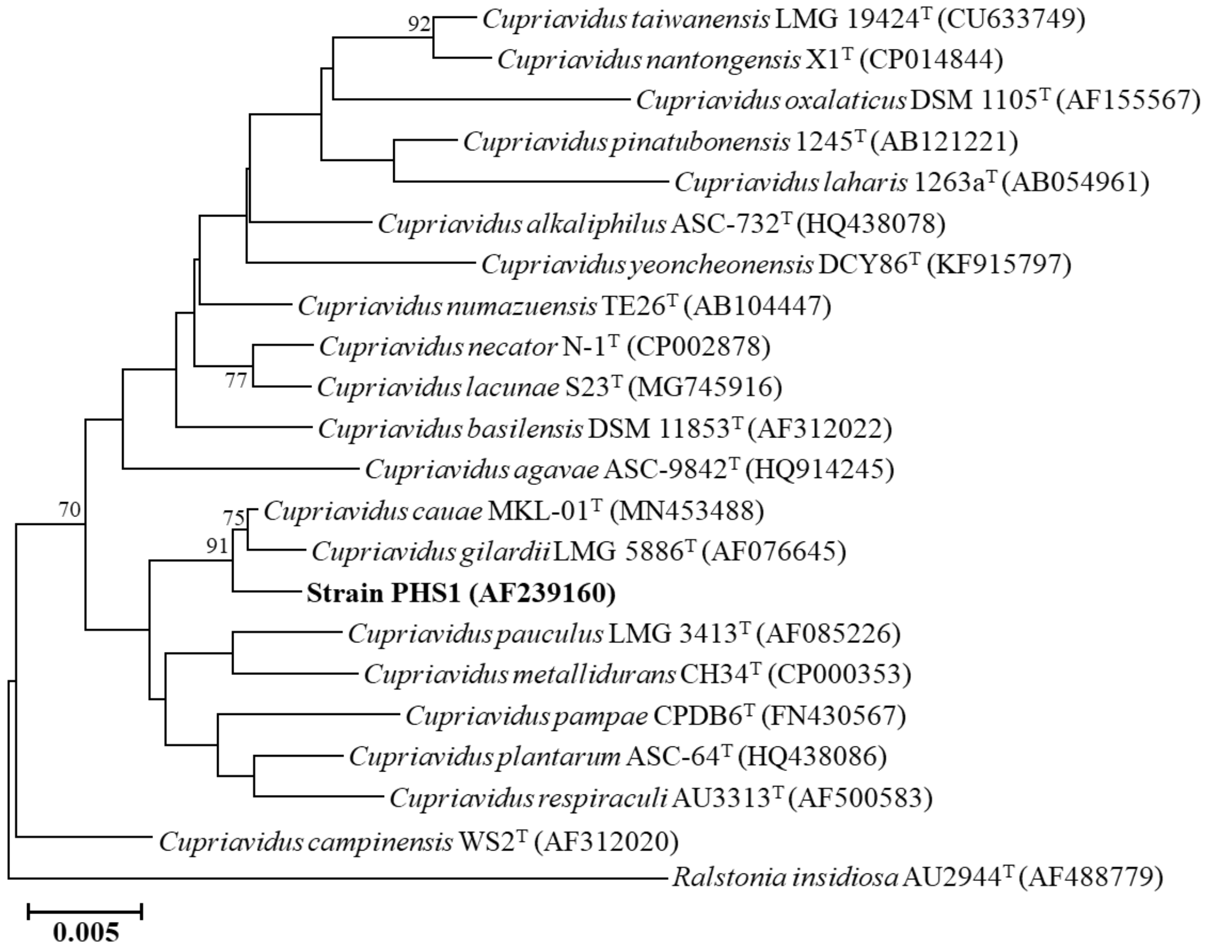

Supplementary Figure S1.

A neighbour-joining tree showing the phylogenetic relationships of strain PHS1 and closely related taxa, based on 16S rRNA gene sequences. Numbers on nodes correspond to bootstrap values for branches (1,000 replicates), shown only bootstrap values over 70%. *R. insidiosa* AU2944 was used as the outgroup. Scale bar, 0.005 changes per nucleotide.

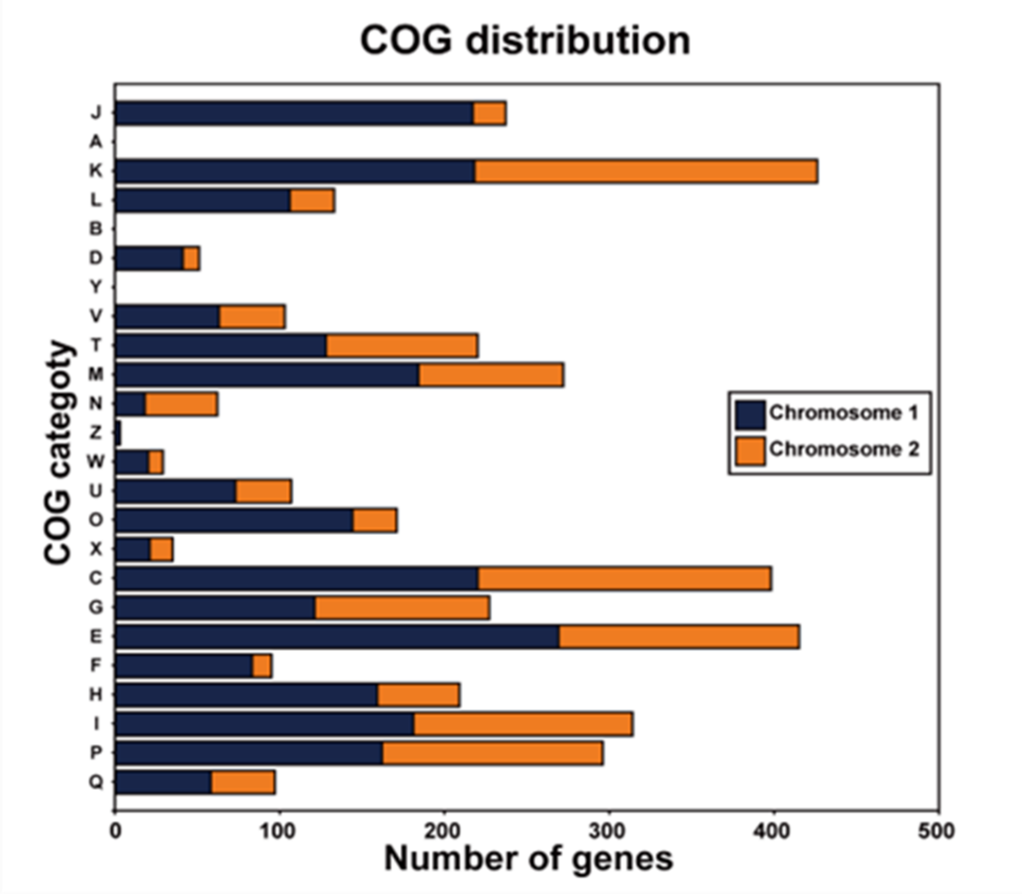

Supplementary Figure S2.

Coding sequence within PHS1 genome were classified into 26 COG categories. Among the COG categories, R (general function only), and S (function unknown) were skipped in figure. COG categories; J: Translation, ribosomal structure and biogenesis, A: RNA processing and modification, K: Transcription, L: Replication, recombination and repair, B: Chromatin structure and dynamics, D: Cell cycle control, cell division, chromosome partitioning, Y: Nuclear structure, V: Defense mechanisms, T: Signal transduction mechanisms, M: Cell wall/membrane/envelope biogenesis, N: Cell motility, Z: Cytoskeleton, W: Extracellular structures, U: Intracellular trafficking, secretion, and vesicular transport, O: Posttranslational modification, protein turnover, chaperones, X: Mobilome: prophages, transposons, C: Energy production and conversion, G: Carbohydrate transport and metabolism, E: Amino acid transport and metabolism, F: Nucleotide transport and metabolism, H: Coenzyme transport and metabolism, I: Lipid transport and metabolism, P: Inorganic ion transport and metabolism, Q: Secondary metabolites biosynthesis, transport and catabolism, R: General Functional Prediction only, and S: Function Unknown.

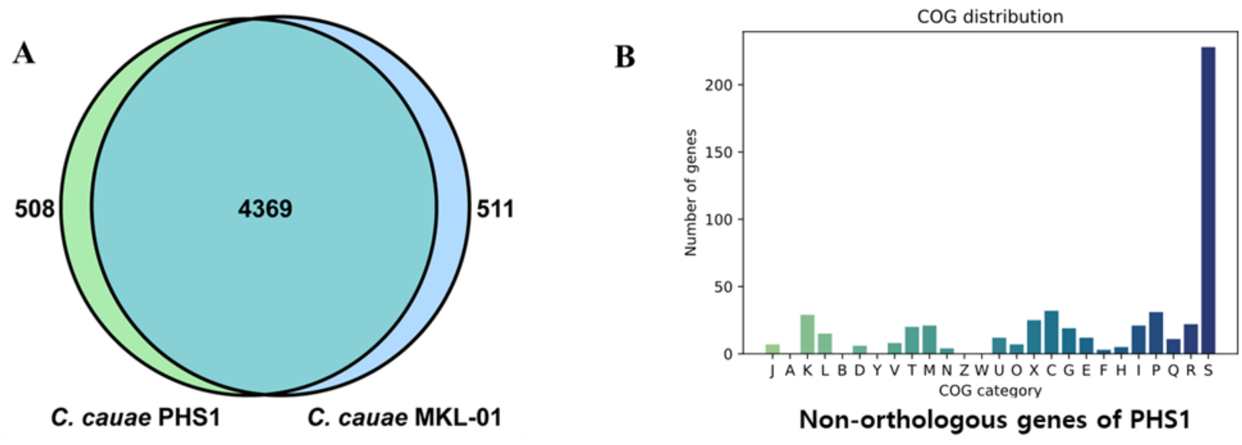

Supplementary Figure S3.

(A) Comparison of orthologous genes in *C. cauae* PHS1 and *C. cauae* MKL-01. **(B)** analysis of COG distribution of non-orthologous genes of PHS1.

Supplementary Table S1

Strains and plasmids used in this study.

Strains and plasmids	Description	Ref.
**Strains**		
PHS1	BTEX-degrading thermotolerant *Cupriavidus cauae* PHS1 isolated from a hot spring in Pohang, Korea.	[8]
E. coli TOP10	F^{-} *mcrA* Δ(*mrr-hsdRMS-mcrBC*) φ80*lacZ*ΔM15 Δ*lacX74 recA1 deoR araD139* (*ara-leu*)*7697 galU galK rpsL*(Str^{R}) *endA1 nupG*	Invitrogen
**Plasmids**		
pSK	pBluescript II SK+, Ap^{r} *lacPOZ'*, T7 and T3 promoter	Stratagene
pSB1	Ap^{r}; 14-kbp *Sau3A1* partial DNA fragment from PHS1 cloned into *BamHI*-digested pSK	This study
pSB1-1	Ap^{r}; *KpnI*-digestion and self-ligation of pSB1	This study
pSB2	Ap^{r}; 6.5-kbp *Sau3A1* partial DNA fragment from PHS1 cloned into *BamHI*-digested pSK	This study

Supplementary Table S2. ANI and DDH comparison of the closest species with strain PHS1.

Strains	ANI value	DDH value
C. cauae MKL-01	98.33	86
C. gilardii W2-2	98.16	85.5
C. gilardii CR3	91.35	45
C. gilardii FDAARGOS_639	91.33	44.9
C. gilardii ATCC 700815	91.40	44.9
C. gilardii CCUG 38401	91.38	44.8
C. gilardii JZ4	91.05	43.7
C. gilardii USM5	90.88	43
C. gilardii Marseille CSURQ4897	90.70	42.9
C. gilardii BF20-02S	90.68	42.6
C. gilardii J11	83.12	27.7

Total 11 subspecies of Cupriavidus cauae and Cupriavidus gilardii were compared with strain PHS1.

ANI, Average nucleotide identity; DDH, DNA-DNA hybridization.
